# Between mandatory and aspirational ethics in nursing codes: a case study of the Italian nursing code of conduct

**DOI:** 10.1186/s12912-024-01697-3

**Published:** 2024-01-10

**Authors:** Stefania Chiappinotto, Michael Igoumenidis, Alessandro Galazzi, Andjela Kokic, Alvisa Palese

**Affiliations:** 1 Department of Medicine, University of Udine, Udine, Italy; 2 Department of Nursing, University of Patras, Patras, Greece

**Keywords:** Aspirational ethics, Mandatory ethics, Codes of conduct, Code of ethics, Nurse, Professionalism

## Abstract

**Background:**

Over the years, national and international nurses’ organisations have drawn up Codes of Conduct and Codes of Ethics. A new differentiation has emerged over time between mandatory and aspirational approaches underlying how nurses can be supported by documents with rules to be respected (mandatory ethics) or by incentives (aspirational ethics). However, to date, no research has applied these approaches to analyse available Codes and to identify which approach are predominantly used.

**Methods:**

In this case study, the Italian Nursing Code of Conduct (NCC), published in 2019, composed of 53 articles distributed in eight chapters, was first translated, and then analysed using a developed matrix to identify the articles that refer to mandatory or aspirational ethics. A nominal group technique was used to minimise subjectivity in the evaluation process.

**Results:**

A total of 49 articles addressing the actions of the individual nurse were considered out of 53 composing the NNC. Articles were broken down into 97 units (from one to four for each article): 89 units (91.8%) were attributed to a unique category, while eight (8.2%) to two categories according to their meaning. A total of 38 units (39.2%) were categorised under the mandatory ethics and 58 (59.8%) under the aspirational ethics; however, one (1.0%) reflected both mandatory and aspirational ethics.

**Conclusions:**

According to the findings, the Italian Professional Body (FNOPI) has issued a modern code for nursing professionals in which an aspirational perspective is dominant offering a good example for other nursing organisations in the process of updating their codes when aimed at embodying an aspirational ethics.

## Background

The term “profession” refers to a special type of occupation or a social role accompanied by an enduring set of normative and behavioural expectations. By and large, professions are self-regulated, and these expectations are usually imprinted in various codes, which amount to private systems of law and are issued by professional organisations [1]. In the specific field of the nursing profession, a rich history of creating and adhering to *codes of conduct* and/or *codes of ethics* has been reported [2], but the distinction between these two different categories of codes is not be clear. Indeed, as Paul Snelling [3] notes, published nursing codes fall into one or other of these categories, but in some codes the functions are confused and/or conflated. Generally, codes of conduct are about nurses’ obligations (standards of practice), whereas codes of ethics are at a higher level and specify how the ideal nurse should be (best practices). In other words, codes of conduct are manifestations of what can be described as *mandatory ethics*, and codes of ethics illustrate the highest standard of ethical practice, for which we can use the term *aspirational ethics* [4].

Within the context of mandatory ethics, nursing professionals are primarily concerned with practising in such a way that protects them from legal and disciplinary sanctions. Therefore, codes of conduct may embody such perspective including prohibited items– underlying forbidden actions and behaviours (negative duties) or a list of acts authorized for practice. From a linguistic perspective, this focus was much more evident in the past, as deontological texts in general and nursing codes in particular would overuse negative phrases, typically beginning with “do not” and “must not”. More positive approaches seem to be promoted at present; for instance, the mandate “do no harm” is now presented as a positive duty in the form of “Nurses facilitate a culture of safety in health care environments” [5]. However, there are those who argue that these inversions do not make a great difference in terms of essence. A positive requirement is just as restrictive as a negative prohibition, and we could replace any given negative rule with a corresponding positive rule, which can be logically and morally equivalent [6]. If there is a duty to tell the truth, it is implied that there is a duty not to lie; if there is a duty to keep patient information confidential, it is implied that there is a duty not to disclose this information, and so on. Inversions are not always as straightforward as the ones presented, but, in many instances, negative and positive duties are simply different sides of the same coin.

Yet positive duties are not only inversions of negative ones: they also refer to acts and attitudes that can be characterised as *supererogatory* – that is, doing more than enough. *Aspirational ethics* ask professionals to exceed the minimum standards of practice and behave in ways that cannot be enforced or covered by codes of conduct. This higher level of ethical behaviour is related to moral values and virtues, and it entails more reflection and discussion than mandates and prohibitions. As such, it integrates professional and personal ethical principles, and it is useful when facing new dilemmas which have never been encountered in practice [4]. A nurse who acts in the context of aspirational ethics strives for the best, even at great personal cost in terms of time, stamina and moral tranquillity. Given that there is no limit on doing more than enough, a conscientious professional could end up engaging in a relentless pursuit of excellence [7], which is simply not attainable.

This remark brings us to the issue of over-demandingness [8], which is the main problematic aspect of aspirational ethics and is especially relevant to nursing practice. Nurses have a duty to display beneficence, but its limits cannot be easily discerned, and no regulatory body can possibly control professionals’ willingness or ability to provide care in a supererogatory way. The nursing profession entails physical and psychological demands, and it is burdened by the continuing problem of under-staffing, so it is often up to individual nurses to decide when they have fulfilled their duties. Depending on context, various factors play a role in setting up these limits at an individual level, such as the nurse’s character and education, the working environment and influence of role-models or even psychological incentives – for instance, describing nurses as “heroes” (in the sense that they do more than their fair share) and heroism as a core nursing value [9]. There are those who argue that the use of heroism stifles meaningful discussion about the duty of care and its limits [10], but we assert that defining limits in care and beneficence is difficult in any case. This can only happen at an individual level and on a case-by-case basis.

One could then ask, what is the point of issuing codes of ethics and including aspirational elements in them? Is there a practical value in stating, for instance, that nurses affirm “the right to universal access to health care for all” [5], as the International Council of Nurses (ICN) does in its Code of Ethics? A nurse can affirm this right, but it certainly represents an ideal that is not feasible and is often not carried out within the context of modern managed care [11]. However, the imperfect implementation of aspirational goals should not discourage the continuing effort to improve nursing professional ethics – or any professional ethics for that matter. Ideal descriptions of nurses’ conduct are important in official texts; they serve as a source of inspiration for new colleagues, and as reminders for those who already practise and may be disillusioned with the health care reality. Besides, codes of ethics have always been aspirational in nature, focusing on virtuous characters who would simply know what their moral duty is, without having to resort to mandates and prohibitions, and would fulfil it to the best of their abilities – acknowledging personal and institutional limits in every case. Aspirational ethics are less definite and open to more exceptions and defences, thus encouraging critical thinking and allowing (but not forcing) professionals to assume greater personal responsibility [12].

Therefore, all professional organisations that follow prohibitive codes are recommended to adopt more aspirational standards when given an opportunity to revise these codes [13]. As noted earlier, nursing codes of conduct and codes of ethics are often merged into single documents, without giving adequate attention to these documents’ titles or to a consistent separation of conduct and ethics throughout the documents [3]. Still, what matters most is whether aspirational elements are included in them, regardless of the title or the terminology used. Their hidden messages [14], shape the practice, ways of thinking and training, as well as the manner that nursing professional behaviours can be judged, criticised, and evaluated. However, to the best of our knowledge, no explorations have been conducted on the available nursing codes of conduct (NCC) or codes of ethics to investigate their hidden perspectives towards an aspirational or a mandatory ethics.

## Methods

### Aim

The aim was to analyse the current Italian NCC [15] by developing and piloting a matrix capable of detecting the aspirational or a mandatory ethics hidden from view. Both the research processes and findings were intended at (a) expanding our knowledge regarding the main trends of the current codes of ethics; (b) providing methodologies and methods for the analysis of nursing codes both for didactic and research purposes; and (c) supporting nursing boards by informing the development or the revision processes of their codes.

### Design

A case study was performed in 2023, relied on the Crowe and colleagues’ methodology [16]. The design was identified according to the bounded-system intrinsic nature of the ethical guidelines in the profession; the unit of analysis was the most recent Italian NCC (Codice Deontologico delle Professioni Infermieristiche) [15], in its role of integrate document containing a set of articles addressing the nursing practice in Italy.

### Unit of analysis and setting

The study was conducted by analysing the Italian NCC [15] developed and approved in 2019 by the National Federation of Nursing Professions Orders (FNOPI), which is a national body representing over 455,000 nurses [17]. It took part in establishing nursing orders at each provincial level. FNOPI was established by law in 1954 [18], and it is considered a subsidiary governmental body addressing the profession development and regulating its practice must follow through the NCC, which has juridical meaning given that all licensed nurses, allowed to practise in Italy after their subscription into the Nursing Order [19]. The current Code, composed of 53 articles distributed in eight chapters [15], was considered relevant to validly achieve the research aims.

### Data collection method

First, we checked to see if the Italian NCC was officially available in the English language, to have a text in a common language given the multinational research team (see authors). Given that no official formal translation was retrieved, its translation in English was provided according to the “forward-only translation” process [20]: first, an expert in language translation, with a master’s degree in European and extra-European languages and literatures (AK), without any background in nursing, provided a first translation. Then, two researchers, educated at the PhD level in the nursing field, checked the translation (SC and AG) and provided changes to better reflect the meaning of each word and statement in the context of nursing care. Then, an additional check was provided by an expert nursing researcher educated at the PhD level (AP). To complete the process, official proof editing was sought from an independent language service, expert in the field of medical translation; the revised draft was checked again by the research team. In case of discrepancies, discussion was planned to solve all disagreements. However, no issues emerged, so this final version was considered in the following research steps [21]. In the meantime, the translation provided was assumed as the official translation by FNOPI and published on the organisation’s web page [22]. The final draft of the translation was then sent to a member of the research team (MI) with a background in nursing ethics and deontology to assess clarity.

The second step was to identify which sections would undergo assessment: the NCC is articulated in 53 articles, and the last four (numbers 50–53) were not taken into consideration because they are devoted to the entire nursing professional board and not to the individual nurse. Given that most articles comprised two or more sentences with different conceptual content, it was decided that the articles would be broken down in units, and then assessed separately. The identification of units was performed by two researchers (SC, AP), before independently and then agreeing upon; the processes resulted in 97 units.

### Data analysis

A nominal group technique (NGT) [23, 24] was established to minimise subjectivity in the evaluations. This methodology is suggested when the research area under investigation is unexplored, when there is no supporting literature or when the information available is contradictory [23]. NGT involves a group of experts [24] in four main steps [24], which we slightly adapted in our case study according to its peculiarity: (a) *silent generation of ideas*, in which each participant writes down their evaluations without comparing them with the others; (b) *round robin*, a round in which all participants express their previous evaluation without, however, discussing them; (c) *discussion*, where evaluations are clarified and similarities confirmed; and (d) *voting/ranking*, or agreeing to establish a consensus in those evaluation with disagreements.

First, an NGT member (MI) developed the analysis matrix (see Table 1) defining the criteria of evaluation for mandatory and aspirational ethics.

**Table 1:** Grid of Nursing Code of Conduct data analysis: Approach, main categories, subcategories and descriptions

Approach	Main categories	Sub-categories	Description
**Mandatory Ethics**	Rules	Entailing legal implications	These rules and their exceptions refer to the *minimum expectations* from practitioners. They are held ethically or legally blameworthy for failing to uphold these rules, but not perceived as praiseworthy for doing so
		Entailing disciplinary implications	
	Specific Exceptions	Descriptive of cases where diversion from a rule is required	
**Aspirational Ethics**	Unspecific Exceptions	Appeal to the practitioner’s moral competence	In contrast to specific exceptions, this type of exception usually demands that practitioners use moral judgment and make up their own exceptions, according to each situation
	Incentives	Related to actions: what would be good for the practitioner to do (skills)	Here lies the main problem with aspirational ethics, in the sense that these *supererogatory actions* may be too demanding
		Related to virtues: what the practitioner’s character should be (attitudes)	In other words, perceived characteristics of the ideal practitioner that may or may not be realistically attainable

**Legend.** We referred to practitioners as the nurse involved in the practice – thus reflecting the target of the NCC articles from 1 to 49

- Mandatory ethics consist of the categories of rules that entail legal implications or rules that entail disciplinary implications and specific exceptions. These rules and their exceptions refer to the minimum expectations from practitioners (in this context, nurses). They are held ethically or legally blameworthy for failing to uphold these rules, but not perceived as praiseworthy for doing so.
- Aspirational ethics consist of the categories of unspecific exceptions – that is, this type of exception usually demands that practitioners (in this context, nurses) use their moral judgment and make up their own exceptions according to each situation and incentives. Incentives can be related to actions, in which lies the main problem with aspirational ethics, in the sense that these supererogatory actions may be too demanding; or they may be related to virtues – that is, to the perceived characteristics of the ideal practitioner that may or may not be realistically attainable.

Based on the data analysis grid (Table 1), agreed upon by the NGT members (see authors), the process began according to the main steps established by Mullen et al. [24]:

- Two researchers (AP and SC) conducted an evaluation of each individual article and unit of the Italian NCC independently, deciding whether each statement fell within the category of mandatory ethics (rules or specific exceptions) or aspirational ethics (unspecific exceptions or incentives).
- During a meeting, both researchers who had conducted the evaluations (AP and SC) presented their results, identifying the areas of common agreement and those of conflict.
- Then, an online NGT meeting was organised in which the two researchers (AP and SC) presented the results achieved to a third researcher (MI). Other members (see authors) were also present. During the meeting, the rationale behind each choice was explained, and the areas of conflict and those of mutual agreement were discussed, in which however the third researcher (MI) sometimes proposed a different categorisation. All researchers were encouraged to write down their motivations after having carefully re-read the evaluation, so the rationale for the choices could be deepened. It was agreed that, in some cases, the units could fall under more than one category; this overlapping was expected, and it was decided not to force a single categorisation, so as not to lose any potential insight.
- During an additional NGT meeting, the remaining conflicting categorisations (7/97 units, 7.2%), were resolved, until consensus was reached. The consensus was qualitatively expressed as the main approach (mandatory or aspirational) embodied in each item of the NCC.

### Rigour

The data analysis was conducted by involving each member of the team and providing independence and cross checking in multiple meetings [25, 26]. Furthermore, considering the required translation into English of the NCC, the process of translation from Italian to English was preliminarily conducted by the Italian team, by involving individuals with different backgrounds; the translation was also checked by the non-native Italian member of the team to assess its clarity [20]. Furthermore, each code article was broken down into units to assess each specific indication: the Italian language is well recognised as having complex construction [27] and to prevent missed elements, each unit was analysed. The integrity of individuals/organisations involved was ensured: no one researcher was involved or had roles in the development of the NCC to ensure an independent evaluation.

## Results

The 49 articles considered of the NCC were broken down into 97 units (from one to four for each article) (Table 2): 89 units (91.8%) reflected a unique category (mandatory or aspirational), while eight (8.2%) were attributed to two categories according to their meaning. A total of 38 units (39.2%) were categorised under the mandatory ethics and 58 (59.8%) under the aspirational ethics; however, one (1.0%) reflected both mandatory and aspirational ethics and was categorised both under *rules entailing disciplinary implication* and *incentives related to actions*.

**Table 2:** Italian Nursing Code of Conduct [15]: Categorization of each Article/unit to emerge the hidden perspectives as aspirational or mandatory ethics

		Mandatory Ethics			Aspirational Ethics		
		Rules		Exceptions		Incentives	
Article	Article sentences divided in units	Entailing legal implications	Entailing disciplinary implications	Specific Exceptions: descriptive of cases where diversion from a rule is require	Unspecific Exceptions: appeal to the practitioner’s moral competence	Related to actions: what would be good for the practitioner to do (skills)	Related to virtues: how the practitioner’s character should be (attitudes)
1	The Nurse is the Health Professional, registered in the Nursing Professions Order,	●					
	who acts in a conscious, autonomous and responsible manner						●
	He or she is supported by a set of values and scientific knowledge.		●				
	Acts as an active agent in the social context to which he or she belongs and in which practices the profession, promoting a culture of caring and safety.					●	
2	The Nurse orients his or her actions to the good of the Assisted Persons, their Family and Community.					●	
	His or her actions are realised and developed in the fields of clinical practice, organisation, education and research.					●	
3	The Nurse treats and cares for the Assisted Person, respecting his or her dignity, freedom, equality, life choices and conception of health and well-being, without any social, gender, sexuality orientation, ethnic, religious or cultural distinction.		●				
	Refrains from any form of discrimination and blame towards all those encountered in his or her work.		●				
4	In his or her professional actions, the Nurse establishes a caring relationship, also using listening and dialogue.					●	
	Ensures that the Assisted Person is never neglected	●					
	by involving, with the consent of the Person concerned, any reference figures as well as other professionals and institutions.					●	
	Relationship time is caring time.		●			●	
5	The Nurse is active in the analysis of ethical dilemmas and contributes to their investigation and discussion.					●	
	Promotes the use of ethical counselling and dialogue, also involving the local Nursing Order.					●	
6	The Nurse undertakes to support the caring relationship even if the Person being cared for manifests ethical conceptions that differ from his or her own.		●				
	If the latter persistently expresses a demand for activities that conflict with personal values or ethical and professional principles of the nurse, he or she ensures continuity of care, taking responsibility for his or her abstention.		●	●			
	The Nurse can make use of the conscience clause, constantly seeking dialogue with the Person being cared for, other professionals and institutions.				●		
7	The Nurse promotes a culture of health by fostering healthy lifestyles and environmental protection from the perspective of health determinants, reducing inequalities						●
	and designing specific educational and informational initiatives for individuals, groups and communities.					●	
8	In his or her various roles, the Nurse is actively involved in the education and professional training of students and the onboarding of new colleagues.					●	
9	The Nurse recognises the value of scientific research and experimentation.						●
	Develops, carries out and participates in research concerning clinical care, organisation and educational, making the results available.					●	
10	The Nurse bases his or her work on knowledge validated by the scientific community and	●					
	updates personal skills through study and research, critical thinking, reflection based on experience and good practice, in order to ensure the quality and safety of activities.		●				
	Plans, conducts and participates in training initiatives and					●	
	fulfils obligations under the Continuing Medical Education programme.		●				
11	The Nurse trains and	●					
	seeks supervision where there are new activities or where there are limited case experience, and in any event whenever the need arises.					●	
12	The Nurse is committed to supporting cooperation with the professionals involved in the care pathway, adopting loyal and collaborative behaviour with colleagues and other professionals.		●				
	Recognises and values their specific contribution in the care process.						●
13	The Nurse acts based on his or her own level of competence	●	●				
	and seeks advice and intervention from experienced nurses or specialists if necessary.					●	
	Advises by making knowledge and skills available to his or her own community and other professional communities and institutions.					●	
	Participates in the care pathway and ensures that the Person cared for has the same information shared with the team, which is necessary for the Person’s life needs and for an informed choice of the proposed care pathways.						●
14	The Nurse who detects a state of alteration of a psycho-physical nature of a professional or other worker in his or her duties, at whatever level of responsibility, shall endeavour to protect and safeguard the Assisted Persons, the profession and the professional in question, including by making the appropriate disclosures.				●		
15	The Nurse ensures that the Person concerned, or the Person he or she refers to, receives accurate, complete and timely information about the person’s state of health, shared with the care team, respecting his or her needs and in a culturally appropriate manner.					●	
	Does not replace other professionals in providing information that is not within his or her competence.	●					
16	The Nurse recognises intra- and inter- professional interaction and integration as fundamental elements for responding to the Person’s needs.						●
17	In the care pathway, the Nurse values and welcomes the person’s contribution, their point of view and emotions and facilitates the expression of suffering.						●
	The nurse informs, involves, educates and supports the Person concerned and, with his or her free consent, the reference persons, in order to encourage adherence to the care pathway and to assess and activate available resources.					●	
18	The Nurse prevents, detects and documents the patient’s pain during the care pathway.					●	
	Operates, applying good practices for the management of pain and related symptoms, while respecting the Person’s wishes.					●	
19	The Nurse guarantees and protects the confidentiality of the relationship with the Person being cared for and the confidentiality of data relating to them throughout their care pathway.	●					
	Collects, analyses and uses data appropriately, limiting him or herself to what is necessary for nursing care, while respecting the rights of the individual and current legislation.	●					
20	The Nurse respects the explicit wish of the Assisted Person not to be informed about his or her state of health.				●		
	If the refused information is necessary to prevent a health risk to third parties, the Nurse shall endeavour to make the Assisted Person aware of the risk and potentially harmful conduct.					●	
21	The Nurse supports the relationship with the Person cared for who is in a condition that limits their expression, through effective communication strategies and modes.					●	
22	Without prejudice to reporting obligations, the Nurse who detects and highlights deprivation, violence or mistreatment of the Person being cared for, takes action to ensure that there is prompt intervention to protect the Person concerned.					●	
23	The Nurse, considering the age and degree of maturity found, shall endeavour to ensure that due consideration is given to the minor’s opinion on curative, care and experimental choices, in order to enable him/her to express his/her wishes.				●		
	When the minor consciously opposes the choice of care, the Nurse works to overcome the conflict.					●	
24	The Nurse provides nursing care until the end of the assisted person’s life.					●	
	Recognises the importance of the caring gesture, shared care planning, palliation, environmental, physical, psychological, relational and spiritual comfort.					●	
	The Nurse supports the Family members and Caregivers of the Person cared for in the final evolution of the illness, in the time of loss and in the grieving phase.					●	
25	The Nurse protects the Assisted Person’s wish to place limits on interventions that he or she believes are not proportionate to his or her clinical condition or consistent with the Person’s conception of quality of life, also expressed in advance by the Person.			●			
26	The Nurse promotes information on blood, tissue and organ donation as an act of solidarity;					●	
	educates and supports those involved in donating and receiving.					●	
27	The Nurse always respects professional secrecy not only out of legal obligation, but out of intimate conviction and as a concrete expression of the relationship of trust with the Person being cared for.	●					
	The death of the Assisted Person does not exempt the Nurse from respecting professional secrecy.	●					
28	In communication, including through information technology and social media, the Nurse behaves with decorum, fairness, respect, transparency and truthfulness;		●				
	he/she protects the confidentiality of Persons and Assisted Persons,	●					
	taking particular care when publishing data and images that may harm individuals, institutions, the decorum and the image of the profession.	●					
29	The Nurse, also through the use of information technology and social media, communicates in a scientific and ethical manner,					●	
	seeking dialogue and discussion in order to contribute to a constructive debate.					●	
30	At the various levels of care, management and training responsibility, the Nurse participates in and contributes to the organisation’s choices, the definition of care, educational and organisational models, the fair allocation of resources and the enhancement of the nursing function and professional role.						●
31	The Nurse contributes to the assessment of the organisational, managerial and logistical context in which the Person being cared for is located in order to protect him/her.					●	
	Formalises and communicates the result of his or her evaluations in order to improve the context itself.					●	
32	The Nurse participates in clinical governance, promotes the best safety conditions for the Person being cared for, adopts procedures for the prevention and management of risks, including infectious ones,					●	
	and actively adheres to operational procedures and methods for analysing events and ways of informing the Persons involved.					●	
33	The Nurse is responsible for the accurate drafting of the clinical documentation for which he/she is responsible,	●					
	emphasising the importance of its completeness and truthfulness also for the purpose of the consent or refusal, knowingly expressed by the Person Assisted, to nursing treatment.					●	
34	Should the organisation request or plan clinical care, management or educational activities that are contrary to the principles, values and standards of the profession, at all levels of responsibility, the Nurse shall report the situation to the competent bodies and					●	
	take action to propose alternative solutions.					●	
35	The Nurse recognises that physical restraint is not a therapeutic act.	●					
	It is exclusively a precautionary measure of an exceptional and temporary nature; it may be implemented by the care team or, in cases of urgent need, even by the Nurse alone if the conditions of necessity are met, in order to protect the safety of the Person being cared for, the other Persons and the workers.			●			
	In any case, physical restraint must be justified and noted in the clinical care documentation, it must be temporary and monitored over time to ascertain whether the conditions that justified its implementation persist and whether it has adversely affected the health of the Assisted Person.	●	●				
36	At the various levels of clinical and managerial responsibility the nurse plans, supervises and verifies, for the safety of the patient, the activities of the nurses’ aides who take part in the care process and are entrusted to him/her.	●	●				
37	Because of his or her high level of professional responsibility,						●
	the Nurse follows the relevant guidelines and good clinical care practices	●	●				
	and ensures their correct application, promoting their continuous updating.					●	
38	The Nurse reports to his or her Nursing Professional Order inappropriate nursing care and assistance activities lacking a sound basis, scientific evidence and validated results.				●		
39	In his or her free professional practice, the Nurse endeavours to ensure that fair competition is respected						●
	and also valorises his or her work through the principle of fair remuneration.					●	
40	The Nurse, with transparency, fairness and in compliance with the regulations in force, formalises with the Assisted Person a special care contract that highlights the adequate and appropriate care needs, what the Person expresses in terms of informed assent/dissent with respect to the proposed treatment, the explicit elements of personal data protection and the elements that make up the professional fee.		●				
41	The self-employed Nurse safeguards the safety and continuity of care of the Persons being cared for by also respecting their own biological and physiological recovery time.		●				
42	The Nurse and the Nursing Professions Orders undertake to ensure that the professional’s actions are free from improper influences and interests as well as undue pressure from third parties, including reference persons, other professionals, companies and associations.	●	●				
43	Any Nurse who finds him or herself in a situation of conflict of interest shall expressly declare it.			●			
44	Nurses and the Nursing Professions Orders counter and denounce the illegal exercise of the nursing profession and undeclared work.	●					
45	The Nurse cares for his or her person and personal decorum.					●	
46	The Nurse exercises the representative function of the profession with dignity, fairness and transparency.					●	
	Uses expressions and adopts behaviours that uphold and promote the decorum and image of the professional community and its institutional actors.						●
	He or she observes the indications of the Nursing Professions Orders in the information and advertising communication.		●				
47	The Nurse complies with the administrative, legal and deontological regulations and requirements that affect the profession, also by following the guidelines of the Nursing Professions Orders.	●	●				
48	The Nurse does not carry out activities of an advisory and expert nature unless he/she possesses the specific skills required by the case.			●			
	In any event, this activity must be carried out in compliance with the deontological principles of the profession, avoiding any conflict of interest and situations in which its independence is limited.				●		
	In the advisory context the Nurse interprets the evidence of the case based on the current scientific knowledge, providing opinions inspired by a prudent assessment of the conduct of the persons involved.					●	
49	The deontological rules contained in this Code of Ethics are binding for all members of the Orders of the Nursing Professions;		●				
	failure to comply with them shall be sanctioned by the Nursing Order, taking into account the voluntariness of the conduct, its severity and any repetition thereof, in contrast with professional decorum and dignity.		●				

**Legend.**: ●, the symbol indicates the chosen category.

Starting with *mandatory ethics* from the category of rules that *entail legal implications*, 20 units (20.6%) reflected this category. These units concerned issues linked to Italian laws and regulations: some examples are those regarding confidentiality (Article 19), person’s rights (Article 19), professional secrecy (Article 27), privacy (Article 28), the use of physical restraints (Article 35) and abusive practices (Article 44). On the other side, the category of rules that *entail disciplinary implications* included 21 units (21.6%), where nurses are expected to demonstrate some professional behaviour (e.g. the care relationship even in case of different ethical conception, Article 6) or some professional values (e.g. dignity, freedom and equality, Article 3). Moreover, regarding the category of *specific exceptions*, only five units (5.1%) were identified: abstention due to conflicting values or ethical and professional principles (Article 6), the limitation of interventions when not proportionate for the condition of the assisted person (Article 25), the use of physical restraint only in exceptional cases (Article 35), issues related to the conflict of interest (Article 43) and the necessity of specific skills in case of consultancy and expert activities (Article 48).

Regarding *unspecific exceptions* related to *aspirational ethics*, six units (6.2%) were included in this category. These concerned complex situations requiring difficult decisions for nurses, such as that regarding the conscience clause (Article 6), the respect of a child’s willingness (Article 23) or that regarding inappropriate nursing care (Article 38). The category of *incentives related to actions* was found to be the richest, including 42 units (43.3%) with various situations and actions that nurses should ideally carry out, such as listening, dialoguing (Article 4); providing educational and informative interventions (Article 7); acting with research in clinical, organisational and educational fields (Article 9); applying good practices for pain management and related symptoms (Article 18); or ensuring person and personal decorum (Article 45). The category of *incentives related to virtues* included 11 units (11.3%) containing the ideal values for an aspiring nurse in his/her profession. Health culture (Article 7), intra- and interprofessional interactions (Article 16) or professional responsibility (Article 37) are some examples in this category.

## Discussion

Our case study investigated the hidden perspectives towards an aspirational or a mandatory ethics embodied in the Italian NCC. The discussion is developed around four main lines: in the context of (a) the overall findings that emerged, (b) the analytical frequencies documented in the two main perspectives (aspirational or mandatory), (c) the research context in the field and (d) the methodological issues.

First, according to the findings, aspirational ethics elements were dominant. Thus, the Italian NCC asks that professionals to exceed the minimum standards of practice and behave in ways that cannot be enforced or covered by codes of conduct: for example, NCC suggests nurses to adopt caring relationships, also using listening and dialogue, or to consider relationship time as caring time (e.g., Article 4). This is a higher level of ethical behaviour related to moral values and virtues, and it entails more reflection and discussion than mandates and prohibitions (e.g., [7]). This finding suggests that the Italian NCC may be considered a good example of Code expressing an aspirational approach; this may help other nursing professional organisations which are due to update their codes and wish to provide a higher-level ethical framework.

Second, according to the frequencies that emerged in the analysis, 38 units fell under the general category of mandatory ethics, and 58 under the category of aspirational ethics (one was categorised in both perspectives). Regarding sentences categorised as mandatory, it is possible to observe how they are often associated with Italian laws or current legislation (e.g. Continuing Medical Education regarding Law 229/1999 [28]; privacy regarding Law 196/2003 [29]). This may explain why these sentences are structured as rules or as specific exceptions to the rules. However, the NCC’s general spirit is oriented towards a higher level, with many positive requirements and few prohibitory items, thus enhancing its aspirational nature. In fact, all the articles concerning communication (e.g. Articles 21, 29) and the relationship with the assisted person (e.g. Article 24), person-centred care (e.g. Article 17), as well as the educational aspects of the professional role (e.g. Article 8), were categorised within the aspirational approach. This suggests an important step for professional development, which no longer places the nurse within a series of activities that he or she must or must not do, but which invites every professional to critically reflect on his or her own actions, activities and choices. To be consistent with this nature, it may be suggested to rename the Italian NCC in the “Code of Ethics and Conduct”. In fact, Snelling [3] argues that there is a significant difference between codes of conduct and codes of ethics, as the former mainly target regulatory functions, whereas the latter describe higher ethical functions. Some professional organisations, such as the Nursing and Midwifery Board of Australia, keep them separated, issuing a Code of Professional Conduct [30, 31] and a Code of Ethics for nurses. However, we see no problem with a mixed, aggregate form, which can be more practical and integrative. All nurses have known colleagues who are inspirational, just as there are some who are less so – for whatever reason. A nursing code should include provisions to cover both cases. It should exemplify supererogatory conduct, and, at the same time, acknowledge that a grounded, regulatory approach is more appropriate for some professionals. In this sense, it should be noted that there exist consciously integrative codes as evidenced by their titles, such as the Nursing Council of Hong Kong Code of Ethics and Professional Conduct [32], and the Nursing and Midwifery Board of Ireland Code of Professional Ethics and Conduct [33]. Future revisions of the Italian Code could use a more integrative title, for reasons of conceptual clarity.

Third, in the context of the research available, this is the first exercise attempting to identify and discern between the mandatory or aspirational nature of a given code in the nursing discipline. Available assessments of various codes [3, 34] have pursued different aims without providing a distinction between mandatory and aspirational. More specifically, Snelling [3] provided a comparative assessment of various nursing codes by considering the ICN Code of Ethics for Nurses, the United Kingdom’s Nursing and Midwifery Council Code, the Nursing and Midwifery Board of Australia Codes of Professional Conduct and of Ethics, the Nursing Council of New Zealand Code of Conduct, the Canadian Nurses’ Association Code of Ethics, the Nursing Council of Hong Kong Code of Ethics and Professional Conduct, the Nursing and Midwifery Board of Ireland Code of Professional Ethics and Conduct and the American Nurses Association Code of Ethics. Snelling [3] has highlighted several significant differences between ethical and conduct codes, which generally correspond to the differences between ethics and law. Our study perspective was different, because the main distinction was between aspirational and mandatory ethics – although mandatory ethics also include legal implications. Other authors have analysed the content and the process of revising nursing codes. For instance, Epstein and Turner [35] described the 2015 revision of the American Nurses Association *Code of Ethics for Nurses with Interpretive Statements*; moreover, Tisdale and Symenuk [34] make a comparative assessment of the nursing codes in Canada from 1957 to 2020 and their relation to human rights in terms of language, positioning and descriptions between different code editions. These approaches are useful, and they can serve to inform nursing scholars and professionals of the value of codes in specific contexts, just as the present research exercise does regarding mandatory and aspirational ethical elements.

Fourth, from the methodological point of view, the analysis resulted with some articles being included in one singular categorisation (e.g. Article 3), while others were categorised under more categories (both rules that entail disciplinary implications and incentives related to actions [Article 4], thus under mandatory and aspirational ethics) and others were under more categories but from the same perspective (e.g. rules that entail legal and disciplinary implications, Articles 35, 37). Despite this being beyond the scope of this research exercise, complex articles including several recommendations with different perspectives (from mandatory to aspirational) may increase the learning complexity of the code among students, as well as the critical analysis of concrete situations. Therefore, the degree of simplicity of each article/element should be debated as an occasion to innovate the code according also to the lessons learnt during the pandemic [36].

Moreover, we have decided to identify categories that were not mutually exclusive: although most units have been classified into only one category (e.g., “Related to virtues: what the practitioner’s character should be (attitudes)”), in eight cases these were included in two categories, mainly under the mandatory ethics as entailing both legal and disciplinary implications. This last peculiarity may reflect the Italian NCC, which has been approved in its value by the law [19], as the basis of professional practice together with the Nursing Profile (defined by the Ministerial Order 739/1994 [37]) and the nursing curriculum as the complex of theoretical and practical competences learnt during the nursing programme. Therefore, the Italian NCC is not only issued by the Nursing Board with deontological purposes but also has legal implications.

An additional reflection concerns the terminology used in the NCC. The most recent Italian Code is called a “Code of Conduct”; however, our research exercise suggests that it contains many elements that can be characterised as aspirational, in the sense that they refer to an ideal nursing professional, and not to someone who just fulfils mandatory duties. From a content point of view, this Code [15] also contains several ethically sensitive terms (e.g., the word “patient” was avoided in favour of “assisted person” [38]) and included articles that concern important issues involving complex ethical choices (e.g., conscience clause, Article 6; physical restraints, Article 35). However, some of these issues, as conscience clause and physical restrain, that are so sensitive from an ethical point of view are still treated with a mandatory approach, and not supporting nurses in higher ethical actions suggesting areas of future debate and improvement.

### Limitations

The research process had several limitations. First, the Italian NCC consists of a series of articles composed of a few to several units [39], and this increased the complexity of the analysis. The Italian language, which is recognised as complex, and the structure of the Italian NCC together suggested breaking the articles up into units, a process that may be not required in the analysis of other codes. For example, the ICN Code of Ethics for Nurses [5] is composed mainly of simple elements or sentences, which would potentially render the analysis easier. Therefore, the data analysis process conducted may require some adaptations when repeated for other nursing codes. Second, only those articles regarding nurse practitioners were analysed, those concerning the professional body were omitted from the analysis (Articles 50–53). Future research exercises should also consider these articles by providing an appropriate framework of analysis. Third, only the most recent NCC was considered; analysing the previous codes (e.g. [40]) established in the Italian context to describe the historical trends may provide meaningful data regarding the developments undertaken by the profession in its ethical reflections and recommendations. Fourth, we performed the categorization of each unit/Article by involving experts in the field with a deep knowledge regarding available laws and rules; however, continuously checking the consistency of the categorization performed to detect changes in the rules and to minimize subjective interpretation is required. Moreover, although we reflected a multicultural perspective involving members from two countries, a wider perspective may be useful to enrich the analysis and the debate (e.g. [41]) by providing, for example, Anglo-Saxon, Germanic, or Scandinavian perspectives.

## Conclusion

This case study was aimed at developing and analysing the Italian NCC with a matrix, to identify of which approach is prevalent in this historical period (mandatory vs. aspirational ethics). Our attempt was to assess whether professional ethics in nursing is based on normative indications (generally negative), or on a higher vision of the profession, that is, aspirational. According to the findings, the Italian Professional Body (FNOPI) has issued a modern code for nursing professionals in which an aspirational perspective is dominant. As a results, it sets a good example for other nursing professional organisations in the process of updating their codes when aimed at embodying an aspirational ethics.

The matrix developed to analyse the code can be further developed to establish an instrument for didactic and professional purposes. Moreover, future studies could use the matrix to compare the current version of the Italian NCC with other codes at the international level, and to detect differences and similarities in their perspectives; moreover, the matrix could be useful to analyse the codes established over time in the same country or across countries to detect changes in the perspectives from mandatory to aspirational or vice versa.

## Data Availability

Data is provided within the manuscript.

## Abbreviations

ICN: International Council of Nurses
NCC: Nursing Code of Conduct
FNOPI: National Federation of Nursing Professions Orders
NGT: Nominal Group Technique
